# Thermoplastic Polymers with Nanosilver Addition—Microstructural, Surface and Mechanical Evaluation during a 36-Month Deionized Water Incubation Period

**DOI:** 10.3390/ma14020361

**Published:** 2021-01-13

**Authors:** Magdalena Ziąbka, Michał Dziadek

**Affiliations:** 1 Department of Ceramics and Refractories, Faculty of Materials Science and Ceramics, AGH University of Science and Technology, 30-059 Krakow, Poland; 2 Department of Glass Technology and Amorphous Coatings, Faculty of Materials Science and Ceramics, AGH University of Science and Technology, 30-059 Krakow, Poland; dziadek@agh.edu.pl

**Keywords:** ABS, PMMA, HDPE, AgNPs, polymeric composites, mechanical and structural properties

## Abstract

Three types of thermoplastic polymers, acrylonitrile butadiene styrene (ABS), polymethyl methacrylate acrylic (PMMA) and high-density polyethylene (HDPE), were enriched with silver nanoparticles (AgNPs) of 0.5 wt.% and 1.0 wt.%, respectively. The polymers and the composites were manufactured via injection molding. Regarding the potential of these polymers as matrices for long-term use as biomaterials, the aim of this study was to examine their stability in the in vitro conditions during a three-year incubation period in deionized water. In this work, microstructural observations were performed, and mechanical properties were assessed. Surface parameters, such as roughness and contact angle, were comprehensively investigated. The microstructural evaluation showed that the silver additive was homogeneously dispersed in all the examined matrices. The 36-month immersion period indicated no microstructural changes and proved the composites’ stability. The mechanical tests confirmed that the composites retained comparable mechanical properties after the silver incorporation. The Young’s modulus and tensile strength increased during long-term incubation. The addition of silver nanoparticles did not alter the composites’ roughness. The contact angle increased with the rising AgNP content. It was also shown that the materials’ roughness increased with the incubation time, especially for the ABS- and HDPE-based materials. The water environment conditions improved the wettability of the tested materials. However, the silver nanoparticles’ content resulted in the contact angle decreasing during incubation. The conducted studies confirmed that the mechanical properties of all the polymers and composites did not deteriorate; thus, the materials may be considered stable and applicable for long-term working periods in aqueous environments.

## 1. Introduction

Synthetic polymers have rapidly entered the medical market due to their advantageous physical, chemical and biological properties, which make them attractive for regenerative medicine, tissue engineering, arthroscopy and joint reconstruction. Polymeric materials may provide functions such as structural support, electrical insulation, the protection of other materials from the body environment, biocompatibility and the delivery of therapeutic drugs [1]. However, the medical applicability of polymers is determined by their mechanical properties and limited by their unintended degradation. Therefore, it is crucial to retain the polymers’ stability and integrity during long-term use under biological conditions. The period when a polymer maintains its designed functionality is the most important measure of its advantageous properties. An example of a polymer with high biological stability retained over time is polymethyl methacrylate acrylic. Polymethyl methacrylate acrylic (PMMA) is not only nonbiodegradable and highly biocompatible but also endowed with great mechanical properties and low acute toxicity. These features make it suitable for medical applications that require permanent and mechanically stable structures. PMMA is commonly used in dentistry as dental cements to produce artificial teeth, orthodontic retainers, dentures, denture bases, obturators, temporary or provisional crowns and repair dental prostheses [2,3]. It is also applied for implant fixation in various orthopedic and trauma surgeries [4]. PMMA is characterized by high values of scratch resistance, a high transparency factor (up to approximately 92% of visible light) and hardness, which makes it applicable in optical products, e.g., lenses and fibers [5]. Another example of a chemically and thermally stable polymer is acrylonitrile butadiene styrene (ABS). Acrylonitrile butadiene styrene is an opaque thermoplastic and an amorphous polymer comprising three monomers: acrylonitrile, butadiene and styrene. It is cheap and endowed with several beneficial properties, including: excellent toughness (even in cold conditions), good dimensional stability, easy process ability, adequate rigidity, high resistance to chemical attack and environmental stress cracking, efficient durability and a low coefficient of thermal expansion [6,7]. Due to its high resistance to a broad range of chemicals and body fluids, along with its hydrolytic stability over time at elevated temperatures, ABS resin is ideal for medical and food-contact fields. ABS is also commonly used to produce respiratory device infusion systems and autoinjection devices [8]. Another example of a thermoplastic polymer applied in biomedicine is High-Density Polyethylene, which is characterized by very good mechanical properties such as high tensile strength, bending and fatigue strength, hardness and appropriate wear resistance, abrasion resistance and a low friction coefficient [9]. HDPE is hydrophobic and has sliding properties that reduce its contact with red blood cells, inhibiting their lysis. As it is nontoxic, HDPE causes neither acute nor chronic allergic reactions and inflammations and has no cancerogenic or mutagenic properties [10]. Existing studies show that HDPE is biocompatible, bioinert and highly corrosion-resistant in the body environment. Therefore, this semicrystalline polymer is widely used in the fabrication of medical devices and implants, e.g., in cranial and facial reconstructions and as an acetabular cup for hip replacements [11].

As described above, due to their unique properties, polymers commonly serve as matrices of biomedical composites used to produce medical equipment, implants and parts of prostheses. In order to enhance the composites’ mechanical properties and improve their bioactive properties, a wide range of modifiers may be applied, such as: polymeric and carbon fibers, carbon nanotubes, boron carbide, boron nitride and graphene oxide nanoparticles [12,13], ceramic particles, hydroxyapatite, tricalcium phosphate, calcium phosphate [14,15], titanium dioxide and bioactive glass [16,17,18]. Noble metals, such as silver and copper, are also applied as nanoparticles (NPs) to improve the bactericidal properties of polymeric composites [19].

With regard to long-term use in the human body, the mechanical properties of implantable thermoplastic polymers are crucial requirements. All implants should be endowed with stability, functionality and long-term reliability without harming the body. Polymers and polymeric composites, during in vitro soaking tests, tend to degrade and lose their mechanical properties due to the influence of the biological environment and temperature. Therefore, thorough investigations should ensure their functionality over a certain period of time [20]. Recently, many studies have described the bactericidal and fungal effects of copper or silver nanoparticles [21]. However, there are few studies devoted to the influence of silver nanoparticles on the mechanical properties of composites over a long research period.

Therefore, in this paper, we described the production process and properties of composites obtained from three polymer matrices filled with different AgNP contents. The purpose of our work was to prove that a low concentration of silver nanoadditives does not necessarily change all the physicochemical properties of thermoplastic polymers. We performed SEM microstructural observation in order to investigate the materials’ long-lasting stability during the in vitro incubation in deionized water for 24 and 36 months. For the mechanical characteristics of the investigated materials, we used a tensile tester machine, and for the evaluation of surface properties, we took into account roughness and wettability.

According to the available literature, and to the best of our knowledge, this is the first study on the in vitro long-term stability of polymer/AgNP composites based on three types of thermoplastic polymers: ABS, PMMA and HDPE.

## 2. Materials and Methods

### 2.1. Material Manufacturing

We produced specimens using three commercially available thermoplastic polymers: acrylonitrile butadiene styrene—Novodur HDM 203FC (INEOS Styrolution, Ludwigshafen, Germany), marked as ABS (Melt Volume-Flow Rate (MVR) = 20 cm^3^ 10 min^−1^, E_t_ = 2550 MPa); polymethyl methacrylate acrylic—Plexiglas SG7 (Altuglas International of Arkema Inc., Dusseldorf, Germany); marked as PMMA (Melt Flow Rate—MFR) = 10 g 10 min^−1^, E_t_ = 2450 MPa); high-density polyethylene—EltexMED HD5226EA-M (INEOS Olefins & Polymers Europe, Koeln, Germany), marked as HDPE (MFR = 26 g 10 min^−1^, E_t_ = 1150 MPa). The composite samples were incorporated with 0.5 and 1.0 wt.% nanosilver (NanoAmor, Katy, TX, USA) of 99.9% purity with an 80 nm particle size and 10.49 g cm^−3^ density. All the samples were prepared via extrusion and injection molding. First, the polymers were dried in a laboratory dryer at 80 °C for 4 h. Then, the polymer granulates were enriched with nanoparticles and homogenized in the plasticizing chamber in the temperature range of 230–240 °C. The next steps were: injecting the homogenized material into a steel molding form, cooling, extracting and shaping as paddles. The process parameters were chosen in accordance with the characteristic datasheet of the polymer manufacturer: injection temperature, 230–240 °C; injection pressure, 80 kg cm^−2^; flow, 80%.

### 2.2. Material Evaluation

All the samples were immersed in deionized water at 37 ± 1 °C for 24 and 36 months. Each material was individually placed in a sterile container in order to avoid contamination. The sample-weight-to-incubation-medium ratio was 1 g:10 mL, which was complied with the ISO 10993-13:2010 Standard [22].

#### 2.2.1. Scanning Electron Microscopy

The Nova NanoSEM 200 scanning electron microscope (FEI, Eindhoven, The Netherlands) with the Genesis XM X-ray microanalysis system (EDAX, Tilburg, Netherlands) featuring the EDAX Sapphire Si(Li) EDX detector was used to perform the microstructural observation and chemical analysis. All the specimens were coated with a carbon layer. The microstructural observations were carried out in low vacuum conditions, using the secondary electron detector (LVD) and the accelerated voltage of 10–18 kV. EDX mapping was performed using a backscattered electron detector (BSE).

#### 2.2.2. Roughness

The arithmetical mean roughness (Ra) of the ABS, PMMA and HDPE polymers and their composites was measured using the contact profilometer HOMMEL-ETAMIC T1000 wave (Jenoptik AG, Jena, Germany). The arithmetical mean roughness values were an average of 10 measurements expressed as the mean ± standard deviation (SD).

#### 2.2.3. Surface Wettability

The sessile drop method with the automatic drop-shape analysis system DSA 10 Mk2 (Kruss GmbH, Hamburg, Germany) was used to determine the static water contact angle of the investigated samples. The temperature and humidity conditions of the measurements were constant. The 0.25 μL Ultra High Quality (UHQ)-water droplets were applied to each pure and dry sample. We calculated the apparent contact angle as an average of 10 measurements and expressed it as the mean ± standard deviation (SD).

#### 2.2.4. Mechanical Test

The tensile strength (σ_M_) and Young’s modulus (E_t_) were measured using the universal testing machine Inspect Table Blue 5 kN with a 5 kN load cell (Hegewald & Peschke, Nossen, Germany). The assumed value of preload force was 1 N and the test speed was 50 mm min^−1^. The samples’ examinations were complied with the EN ISO 527-1 Standard [23]. For all the investigated samples, 10 measurements were taken and expressed as the mean ± standard deviation (SD).

#### 2.2.5. Statistical Analysis

In this article, the results were analyzed using the one-way analysis of variance (ANOVA) with Duncan’s post hoc tests, which were performed with the Statistica 10 (StatSoft^®^, Tulsa, OK, USA) software. The results were considered statistically significant when *p* < 0.05.

## 3. Results and Discussion

**Figure 1:**
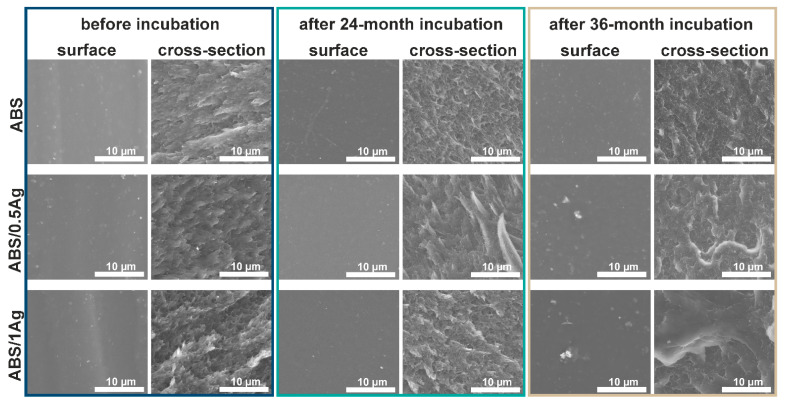
SEM images of the surface and cross-section of acrylonitrile butadiene styrene (ABS) and ABS-based composites modified with 0.5 wt.% and 1.0 wt.% silver nanoparticles (AgNPs) before and after the 24- and 36-month incubation periods in deionized water.

**Figure 2:**
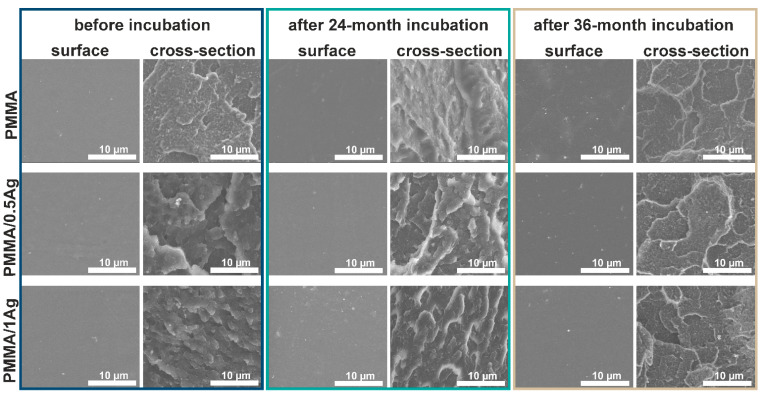
SEM images of surface and cross-section of polymethyl methacrylate acrylic (PMMA) and PMMA-based composites modified with 0.5 wt.% and 1.0 wt.% AgNPs before and after the 24- and 36-month incubation periods in deionized water.

**Figure 3:**
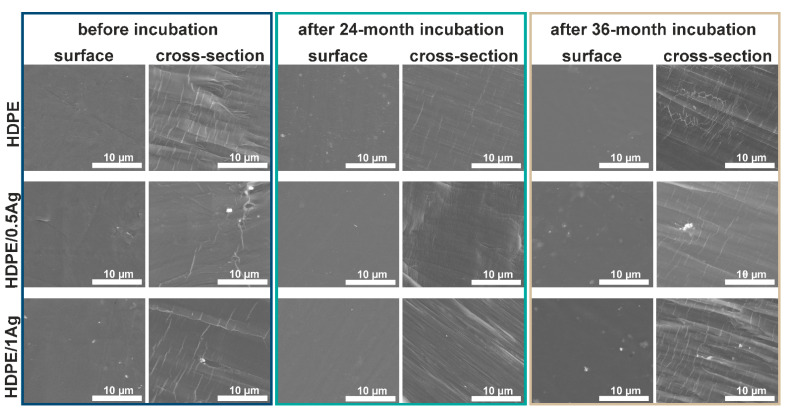
SEM images of surface and cross-section of high-density polyethylene (HDPE) and HDPE-based composites modified with 0.5 wt.% and 1.0 wt.% AgNPs before and after the 24- and 36-month incubation periods in deionized water.

Scanning electron microscopy was used to investigate changes in the surface and cross-section of the samples immersed in deionized water for two and three years. The microstructural observations of polymers ABS (Figure 1), PMMA (Figure 2) and HDPE (Figure 3)) and their silver composites showed that all the samples had smooth surfaces, i.e., free of cracks, holes or any other defects. The silver nanoparticles were homogeneously dispersed throughout the matrix volume, which was proven by the EDX analysis (data are shown in the Supplementary Materials, Figure S1). However, individual micrometric silver agglomerates appeared both on the surface of the materials and in the composites’ cross-sections. This phenomenon was especially noticeable for the composites with 1.0 wt.% AgNPs. Grigoriadou et al. [24] revealed similar observations. No significant changes were observed in the samples’ microstructure after 24 and 36 months of the in vitro incubation. However, the silver nanoparticles were more visible on the surfaces investigated after incubation, which might be connected with the nanoparticles leaching from the polymer matrix and the silver ions’ release. Such behavior was described in previous works [25,26]. It was observed that the cross-sections of polymers and composites on the ABS and PMMA matrices had an appearance characteristic of amorphous polymers (lamella-like structure and ductility) [27]. The microstructure of the HDPE polymer cross-section was relatively flat and smooth, with visible areas of the microfibril existence [28].

The microstructural observation revealed no cracks, indicating structural changes in the polymer matrices’ surface or the cross-sections. Therefore, it might be concluded that no degradation occurred in the investigated materials during incubation.

**Figure 4:**
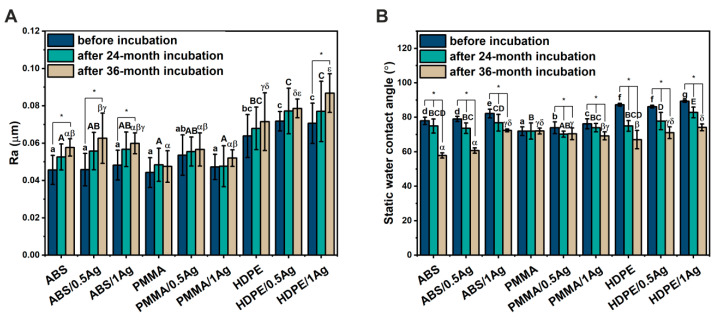
Arithmetical mean roughness (Ra) (**A**) and static water contact angle (**B**) of polymers and composites modified with 0.5 wt.% and 1.0 wt.% AgNPs. Statistically significant differences (*p* < 0.05) between different materials before as well as after the 24- and 36-month incubation periods are indicated by subsequent lowercase and uppercase Latin letters and Greek letters, respectively. Different letters indicate statistically significant differences. Statistically significant differences (*p* < 0.05) between materials before and after the 24- and 36-month incubation periods are indicated by asterisks (*).

The materials’ surface roughness was assessed on the basis of the Ra parameter value before and after the 24- and 36-month incubation periods in deionized water (Figure 4A, Table 1). For all the materials’ groups (ABS, PMMA, HDPE), the presence of Ag nanoparticles did not significantly affect the composites’ surface roughness. The ABS and PMMA materials were characterized by similar roughness values, and the HDPE materials showed higher values of the tested parameter. The surface roughness values of all the materials increased during incubation in deionized water (by 6–12% after the 36-month incubation period), with statistically significant differences observed only for the ABS-based materials (by 24–37% after the 36-month incubation period) and the HDPE/1Ag composite (by 23% after the 36-month incubation period). Importantly, the nanoparticles’ presence in the individual polymer matrices did not significantly affect the surface roughness changes during the incubation time compared to the unmodified materials. Both prior to incubation and after 36 months, the ABS and PMMA materials were characterized by the Ra values lower than the HDPE materials ones. Moreover, all the materials showed low surface roughness (Ra in the range of 0.035–0.1 μm) before and after long-term incubation.

The materials’ surface wettability before and after 4- and 36-month incubation periods in deionized water was evaluated via the static contact angle measurement (Figure 4B, Table 2). The higher concentration of Ag nanoparticles (1 wt.%) in all the polymer matrices resulted in a statistically significant increase in the contact angle. Even the 0.5% addition of nanoparticles to PMMA-based materials led to a significant contact angle increase. Moreover, the contact angle with regard to the matrix material increased as follows: PMMA < ABS < HDPE. All the materials revealed contact angle values below 90°, which indicated their hydrophilic nature. Long-term incubation in deionized water significantly improved the materials’ hydrophilicity; the only exception was the PMMA polymer material, revealing no changes in surface wettability. After incubation, the contact angle values decreased more significantly for the ABS materials (by 12–26% after the 36-month incubation period) and the HDPE materials (by 17–23% after the 36-month incubation period) in comparison to the PMMA materials (by 5–9% after the 36-month incubation period). Additionally, the ABS and HDPE materials revealed that the Ag nanoparticles’ presence in the composite matrix slightly lowered the contact angle during incubation, which was particularly noticeable for the ABS samples.

The variations in surface wettability of the polymer-based materials during long-term incubation in deionized water may indicate the surface chemical changes. During prolonged immersion in the aqueous environment, more hydroxyl or carboxyl polar groups may be incorporated onto the surfaces of polymeric materials, improving their surface wettability [29,30]. On the other hand, surface wettability is strongly influenced by its topography. According to the Wenzel and Cassie models, higher surface roughness improves the hydrophilicity of hydrophilic surfaces and the hydrophobicity of hydrophobic surfaces [31]. As all the materials prior to incubation were hydrophilic, the reduced contact angle during incubation correlated with the increased surface roughness. The lowest changes in surface wettability during incubation were observed for the PMMA materials, which was consistent with the lowest Ra-value variations for these materials.

**Figure 5:**
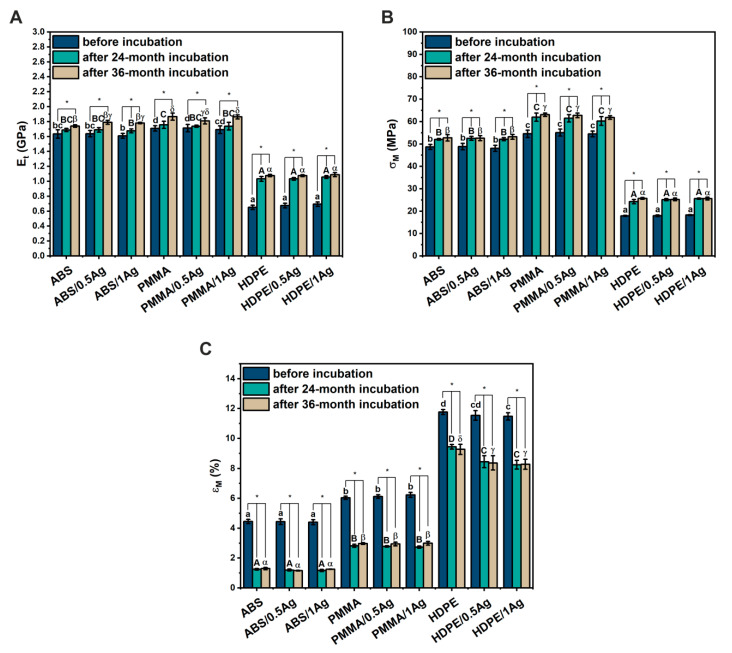
Young’s modulus (**A**), tensile strength (**B**) and elongation at maximum force (**C**) of polymers and composites modified with 0.5 wt.% and 1.0 wt.% AgNPs. Statistically significant differences (*p* < 0.05) between different materials before and after the 24- and 36-month incubation periods are indicated by subsequent lowercase and uppercase Latin letters and Greek letters, respectively. Different letters indicate statistically significant differences. Statistically significant differences (*p* < 0.05) between materials before and after the 24- and 36-month incubation periods are indicated by asterisks (*).

The materials’ mechanical properties—Young’s modulus (Et), tensile strength (σM) and elongation at maximum force (εM)—were assessed by the static tensile test before and after the 24- and 36-month incubation periods in deionized water (Figure 5, Table 3, Table 4 and Table 5). All the polymer matrices revealed that the addition of Ag nanoparticles did not significantly affect the Et, σM and εM parameters. The only exception was the HDPE/1Ag composite whose elongation value at maximum force decreased in comparison to the HDPE polymer material. Taking into account the matrix material, the values of the Et and σM parameters increased as follows: HDPE << ABS < PMMA. The Young’s modulus values of the individual groups corresponded to the elongation at maximum force values and increased as follows: ABS < PMMA << HDPE. Our results confirmed the literature data by Yu et al. [32], who investigated how the increasing concentration of copper nanoparticles influenced the mechanical properties of HDPE-based composites. They noticed that all the nanocomposite monofilaments had an elastic modulus higher than pure HDPE monofilaments and the nanocomposite monofilaments modulus first increased and then decreased with increasing CuNP content. Such behavior correlated with the effective nanoparticle dispersion and interfacial particles/matrix adhesion so that the mobility of polymer chains was restricted under loading [33]. The significant increase in the Young’s modulus and tensile strength for the HDPE/Ag composites might correlate with the results proven by Su et al. [34]. The authors carried out mechanical evaluations of polyethylene and its composites with silver nanoparticles at AgNP weight fractions of 1.05 wt.% and 3.10 wt.%. They proved that the silver nanoparticles could significantly improve the polyethylene Young’s modulus and tensile strength due to improvements in the local density and strength near the AgNP surface in the range of 12 Å.

Long-term incubation of materials in deionized water significantly influenced their mechanical properties. Similar behavior was observed in our previous studies [35,36,37]. The Young’s modulus and tensile strength values increased along with the incubation time. On the other hand, the elongation at maximum force values decreased significantly for all the tested materials. It is worth mentioning that the presence of nanoparticles in individual polymer matrices did not significantly change the mechanical parameters during incubation compared to the unmodified materials. The only exceptions were the HDPE composites (HDPE/0.5Ag and HDPE/1Ag), which revealed that the εM value decreased after the 24- and 36-month incubation periods. Having been incubated, the E_t_ values of the HDPE materials (by 57–65% after the 36-month incubation period) significantly increased in comparison to the PMMA materials (by 6–10% after the 36-month incubation period) and ABS (by 6–11% after the 36-month incubation period). On the other hand, when analyzing the εM changes during incubation, a much greater decrease was observed for PMMA (by 51–52% after the 36-month incubation period) and ABS (by 71–74% after the 36-month incubation period) compared to HDPE (by 21–28% after the 36-month incubation period).

## 4. Conclusions

In this article, we studied how silver nanoparticle content affected the mechanical properties of ABS/AgNP, PMMA/AgNP and HDPE/AgNP nanocomposites. For this purpose, the nanocomposite samples were prepared via injection molding and thoroughly examined. The SEM images confirmed the AgNP’s homogenous dispersion in the polymer’s matrix, with noticeable agglomerates in the case of the 1.0 wt.% silver additive. Then, with the results of the tensile tests, we investigated the effects of nanoparticle concentration on the mechanical properties. The results showed that the addition of 0.5 wt.% and 1.0 wt.% silver nanoparticles did not adversely affect the tensile strength, Young’s modulus and elongation at break values of the PMMA and ABS samples. The only exception was the HDPE matrix with both 0.5 wt.% and 1.0 wt.% AgNPs, of which the value of elongation at break decreased. Neither the tensile strength nor the Young’s modulus values of the investigated materials deteriorated due to incubation. It was also shown that these parameters increased with the incubation time, and the highest increase was observed for the HDPE/AgNP composites. The improved mechanical properties confirmed that such composites may be used in biomedical applications over a long period of time. Furthermore, despite the rising nanoparticle content, no significant changes in the roughness value were observed while the contact angle value increased. The samples’ incubation improved the roughness and hydrophilicity values of the tested materials. Our results indicate that such composites materials are suitable for biomedical applications, particularly for manufacturing small implants, such as middle ear implants, where the key factors are bactericidal properties and stable mechanical parameters.

## Figures and Tables

**Table 1:** Results of the arithmetical mean roughness of polymers and composites modified with 0.5 wt.% and 1.0 wt.% AgNPs.

Material	Arithmetical Mean Roughness Ra (µm)		
	Before Incubation	After 24-Month Incubation	After 36-Month Incubation
ABS	0.046 ± 0.008	0.053 ± 0.007	0.057 ± 0.005
ABS/0.5Ag	0.046 ± 0.009	0.056 ± 0.010	0.063 ± 0.013
ABS/1Ag	0.048 ± 0.008	0.057 ± 0.009	0.060 ± 0.006
PMMA	0.044 ± 0.008	0.048 ± 0.008	0.048 ± 0.008
PMMA/0.5Ag	0.054 ± 0.011	0.055 ± 0.008	0.057 ± 0.009
PMMA/1Ag	0.047 ± 0.007	0.048 ± 0.011	0.052 ± 0.004
HDPE	0.064 ± 0.012	0.068 ± 0.011	0.072 ± 0.016
HDPE/0.5Ag	0.072 ± 0.005	0.077 ± 0.012	0.079 ± 0.005
HDPE/1Ag	0.071 ± 0.011	0.077 ± 0.017	0.087 ± 0.010

**Table 2:** Results of the static water contact angle of polymers and composites modified with 0.5 wt.% and 1.0 wt.% AgNPs.

Material	Static Water Contact Angle (°)		
	Before Incubation	After 24-Month Incubation	After 36-Month Incubation
ABS	78.0 ± 2.1	74.9 ± 4.1	57.9 ± 1.5
ABS/0.5Ag	79.1 ± 1.5	73.7 ± 3.0	60.7 ± 1.5
ABS/1Ag	82.2 ± 2.5	76.9 ± 4.8	72.4 ± 0.8
PMMA	71.9 ± 2.6	72.0 ± 4.7	72.0 ± 1.7
PMMA/0.5Ag	73.9 ± 3.4	70.3 ± 1.7	70.4 ± 3.4
PMMA/1Ag	76.2 ± 2.9	74.0 ± 2.4	69.2 ± 2.4
HDPE	87.3 ± 0.9	75.0 ± 3.1	67.0 ± 5.3
HDPE/0.5Ag	86.2 ± 0.8	77.8 ± 5.0	71.0 ± 3.4
HDPE/1Ag	89.4 ± 0.7	82.8 ± 3.1	74.1 ± 1.9

**Table 3:** Results of the Young’s modulus of polymers and composites modified with 0.5 wt.% and 1.0 wt.% AgNPs.

Material	Young Modulus E_t_ (GPa)		
	Before Incubation	After 24-Month Incubation	After 36-Month Incubation
ABS	1.63 ± 0.06	1.69 ± 0.02	1.74 ± 0.02
ABS/0.5Ag	1.64 ± 0.04	1.69 ± 0.03	1.79 ± 0.03
ABS/1Ag	1.61 ± 0.03	1.68 ± 0.03	1.78 ± 0.01
PMMA	1.71 ± 0.04	1.76 ± 0.05	1.87 ± 0.05
PMMA/0.5Ag	1.71 ± 0.05	1.74 ± 0.01	1.81 ± 0.04
PMMA/1Ag	1.69 ± 0.05	1.74 ± 0.05	1.86 ± 0.03
HDPE	0.65 ± 0.03	1.03 ± 0.03	1.08 ± 0.01
HDPE/0.5Ag	0.68 ± 0.03	1.03 ± 0.02	1.08 ± 0.01
HDPE/1Ag	0.69 ± 0.03	1.06 ± 0.02	1.09 ± 0.03

**Table 4:** Results of the tensile strength of polymers and composites modified with 0.5 wt.% and 1.0 wt.% AgNPs.

Material	Tensile Strength σ_M_ (MPa)		
	Before Incubation	After 24-Month Incubation	After 36-Month Incubation
ABS	48.70 ± 1.13	52.06 ± 0.36	52.77 ± 1.42
ABS/0.5Ag	48.83 ± 1.43	52.51 ± 0.90	52.60 ± 1.14
ABS/1Ag	48.05 ± 1.33	52.12 ± 0.67	53.19 ± 1.07
PMMA	54.54 ± 1.64	61.99 ± 1.81	63.05 ± 0.76
PMMA/0.5Ag	55.09 ± 1.57	61.49 ± 1.64	62.80 ± 1.04
PMMA/1Ag	54.53 ± 1.27	60.26 ± 1.91	61.80 ± 0.79
HDPE	17.88 ± 0.23	24.24 ± 0.97	25.72 ± 0.38
HDPE/0.5Ag	17.94 ± 0.34	25.13 ± 0.50	25.23 ± 0.59
HDPE/1Ag	18.26 ± 0.18	25.60 ± 0.28	25.62 ± 0.66

**Table 5:** Results of the elongation at maximum force of polymers and composites modified with 0.5 wt.% and 1.0 wt.% AgNPs.

Material	Elongation at Maximum Force ε_M_ (%)		
	Before Incubation	After 24-Month Incubation	After 36-Month Incubation
ABS	4.40 ± 0.16	1.17 ± 0.07	1.25 ± 0.01
ABS/0.5Ag	6.04 ± 0.11	2.82 ± 0.10	2.97 ± 0.06
ABS/1Ag	6.13 ± 0.12	2.78 ± 0.05	2.94 ± 0.13
PMMA	6.23 ± 0.17	2.73 ± 0.07	2.99 ± 0.13
PMMA/0.5Ag	11.77 ± 0.17	9.44 ± 0.16	9.27 ± 0.34
PMMA/1Ag	11.54 ± 0.33	8.45 ± 0.40	8.37 ± 0.48
HDPE	11.48 ± 0.25	8.25 ± 0.29	8.28 ± 0.33
HDPE/0.5Ag	4.40 ± 0.16	1.17 ± 0.07	1.25 ± 0.01
HDPE/1Ag	6.04 ± 0.11	2.82 ± 0.10	2.97 ± 0.06

## Data Availability

The data presented in this study are available on request from the corresponding author.

## Supplementary Materials

The following are available online at https://www.mdpi.com/1996-1944/14/2/361/s1, Figure S1: Mapping analysis of ABS*, PMMA and HDPE containing 1 wt.% of silver nanoparticles (AgNPs). *ABS mapping do not show N distribution.
