# Supplementary material for: Thermoplastic Polymers with Nanosilver Addition—Microstructural, Surface and Mechanical Evaluation during a 36-Month Deionized Water Incubation Period
Source: Materials (Basel). 2021 Jan 13;14(2):361. doi: 10.3390/ma14020361 (PMC7828428; doi:10.3390/ma14020361)
Supplement: Supplementary file 1 [file materials-14-00361-s001.pdf]

materials

MDPI

Supplementary Materials

Thermoplastic Polymers with Nanosilver Addition—Microstructural, Surface and Mechanical Evaluation during a 36-Month Deionized Water Incubation Period

Magdalena Ziąbka [1,*] and Michał Dziadek [2]

[1] Department of Ceramics and Refractories, Faculty of Materials Science and Ceramics, AGH University of Science and Technology, 30-059 Krakow, Poland
[2] Department of Glass Technology and Amorphous Coatings, Faculty of Materials Science and Ceramics, AGH University of Science and Technology, 30-059 Krakow, Poland; dziadek@agh.edu.pl
* Correspondence: ziabka@agh.edu.pl; Tel.: +48-012-617-2523

Citation: Ziąbka, M.; Dziadek, M. Thermoplastic Polymers with Nanosilver Addition—Microstructural, Surface and Mechanical Evaluation during a 36-Month Deionized Water Incubation Period. *Materials* **2021**, *14*, 361. https://doi.org/10.3390/ma14020361

Received: 3 December 2020
Accepted: 11 January 2021
Published: date

Publisher's Note: MDPI stays neutral with regard to jurisdictional claims in published maps and institutional affiliations.

Copyright: © 2021 by the authors. Submitted for possible open access publication under the terms and conditions of the Creative Commons Attribution (CC BY) license (http://creativecommons.org/licenses/by/4.0/).

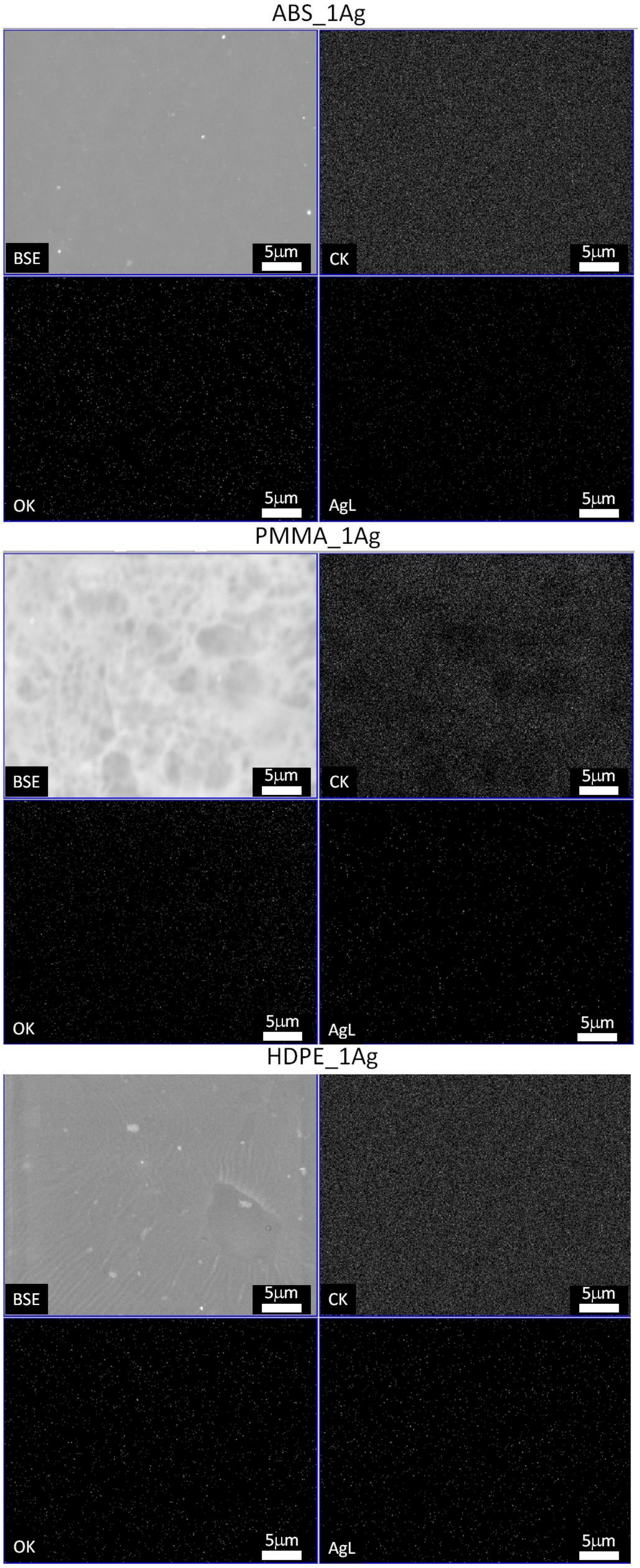

Figure S1. Mapping analysis of ABS*, PMMA and HDPE containing 1 wt.% of silver nanoparticles (AgNPs).*ABS mapping do not show N distribution.
